# Dendritic Spines and Development: Towards a Unifying Model of Spinogenesis—A Present Day Review of Cajal's Histological Slides and Drawings

**DOI:** 10.1155/2010/769207

**Published:** 2011-03-13

**Authors:** Pablo García-López, Virginia García-Marín, Miguel Freire

**Affiliations:** Department of Molecular, Cellular and Developmental Neurobiology, Instituto Cajal, CSIC, Avenida Doctor Arce 37, 28002 Madrid, Spain

## Abstract

Dendritic spines receive the majority of excitatory connections in the central nervous system, and, thus, they are key structures in the regulation of neural activity. Hence, the cellular and molecular mechanisms underlying their generation and plasticity, both during development and in adulthood, are a matter of fundamental and practical interest. Indeed, a better understanding of these mechanisms should provide clues to the development of novel clinical therapies. Here, we present original results obtained from high-quality images of Cajal's histological preparations, stored at the Cajal Museum (Instituto Cajal, CSIC), obtained using extended focus imaging, three-dimensional reconstruction, and rendering. Based on the data available in the literature regarding the formation of dendritic spines during development and our results, we propose a unifying model for dendritic spine development.

## 1. Introduction

**Figure 1:**
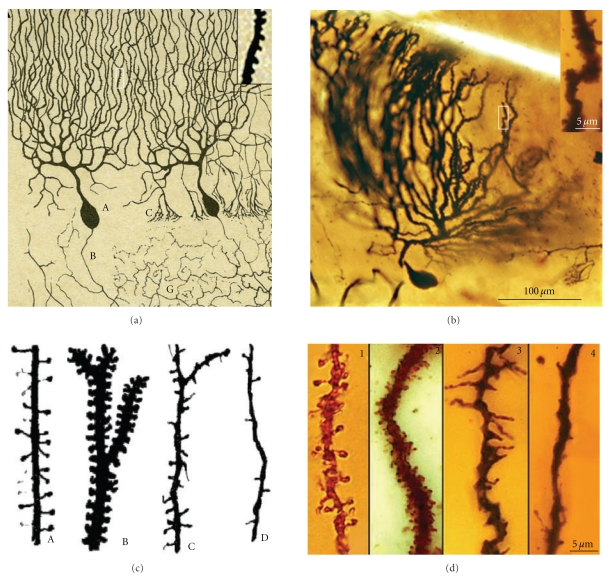
(a) Represents the first Cajal scientific drawing showing dendritic spines from a Purkinje cell of the hen, 1888. Inset shows dendritic spines digitally enlarged of the boxed region. (b) Purkinje cell and dendritic spines (inset) of the adult bird cerebellum taken from a Cajal's histological preparation stained following the Golgi method. (c) drawing by Cajal [5] showing dendritic spines of pyramidal (A), Purkinje (B), basket (C) and Golgi cells (D). (d) dendritic spines and filopodia taken from Cajal histological preparations, and Golgi impregnation; 1, dendritic spines, pyramidal cell, parietal cortex, one-month-old human; 2, dendritic spines, Purkinje cell, adult cat cerebellum; 3, dendritic filopodia and spines, basket cell, cerebellum, of 17-day-old dog; 4, Golgi cell dendrite, cerebellum, of 17-day-old dog.

Dendritic spines were first discovered by Cajal ^1^ in 1888 when studying the dendrites of Purkinje cells in hens (Figures 1(a) and 1(b)):

*“...la superficie... aparece erizada de puntas ó espinas cortas...” *(Cajal, 1888) [1]. ^2^

**Figure 2:**
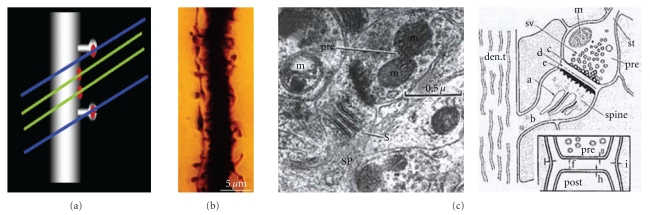
(a) 3D-model showing the physiological interpretation of the dendritic spines by Cajal that evolved from a role in canalizing the nervous fibers for contacting the dendrite (see [3], in green) to a connective function (see [4–7]; in blue). (b) Dendritic spines of an apical pyramidal trunk supposedly contacting an axon, Cajal histological preparation, Golgi impregnation. (c) Electron microscopy and scheme of dendritic spines as postsynaptic elements, gray [8].

Cajal then extended the study of dendritic spines to many different types of cells and species (Figures 1(c) and 1(d), see [2]) suggesting a functional role for the dendritic spines. His concept of these structures evolved from their role in canalizing nerve fibers towards dendrite profiles (see [3], Figure 2(a) green lines) to a connective function (see [4], Figures 2(a), blue lines and 2(b)), incrementing the surface area of neurons and/or extending the dendrite to make contact with distant nerve fibers [5–7]. The role of dendritic spines in connecting nerve fibers was later demonstrated by means of electron microscopy (see [8], Figure 2(c)), which led to the general agreement that the majority of excitatory connections in the cerebral cortex are established at dendritic spines [9, 10], even though excitatory connections can also end on dendritic shafts [11, 12]. Many authors have contributed to the knowledge of the dendritic spines using light microscopy, electron microscopy and three-dimensional (3D) reconstructions on serial ultrathin sections; studying plastic changes, distribution, pathological changes, and so forth, (see [13]).

How axodendritic contacts are constructed during development still remains unclear. Understanding how dendritic spines emerge and develop, and the relationship between synaptogenesis and spinogenesis, would provide insight into: (i) the correct wiring of brain circuits during development, including the selective formation and retention of synaptic contacts between neurons; (ii) the timing of the appearance of dendritic spines in relation to the initial establishment of axodendritic contacts; (iii) the mechanisms to arrange new connections in response to experience in the adult animal; and (iv) the underlying mechanisms of severely altered synaptogenesis and spinogenesis that occur in various disease states (fragile X syndrome, Down's syndrome, etc.). 

Different models of both spinogenesis and synaptogenesis have been proposed, and we will principally examine the former [14], discussing the different factors thought to be implicated in spinogenesis (genetic factors, dendrite-axon interactions, nervous activity, etc.). Then, we will review studies on the development of dendritic spines in the cerebellum (Purkinje cells, granule cells, basket cells, and Golgi cells), cerebral cortex (pyramidal cells, double bouquet cells, basket cells) and olfactory bulb (granule cells), finally proposing a unifying model of spinogenesis.

 Histological slides produced by Santiago Ramón y Cajal were employed. These slides, stored at the Cajal Institute, contain sections of the developing cerebellum, cerebral cortex and olfactory bulb, which allowed us to follow the evolution of dendritic spines at different points in the development of different animal species, including birds, the cats, dogs, humans, and rabbits. The application of the so-called “ontogenic method” by Cajal constitutes, from our point of view, one of the most important reasons for his success in Neuroscience. Instead of studying the complexity of the adult brain, he focused first on the nervous system of lower animals, embryos and young higher animals. Thanks to this approach, many of his histological slides are from different species, which allowed us to readily follow the evolution of filopodia and dendritic spines. We have obtained very high-quality extended focus images, 3D-reconstructions and rendered images that we have correlated with recent live imaging studies. 

## 2. Models of Spinogenesis

**Figure 3:**
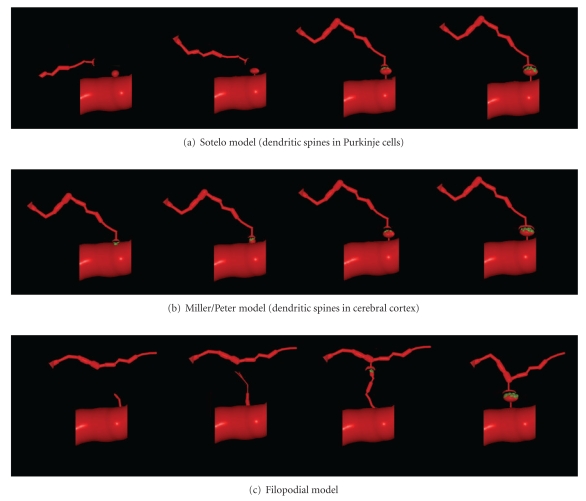
Models of spinogenesis based on the Figure  3 from Yuste and Bonhoeffer [14]. (a) *Sotelo model*: dendritic spines emerge from dendritic trees by an autonomous cellular program, independently of the axonal terminals. (b) *Miller/Peters model*: dendritic spines are induced by axonal terminals on dendrites by a successive outgrowth through a sessile spine to a pedunculated one. (c) **Filopodial model **: filopodia become dendritic spines interacting with axons. Synaptic contacts in green.

Different methods have been used to study the formation of nervous connections during development. Ultrastructural data have provided insight into the fine morphology of synapses, whereas live-imaging techniques shed light on the dynamics of both the presynaptic terminal and the postsynaptic dendrite, as well as on spinogenesis and its relation to synaptogenesis. These data suggest that different mechanisms underlie spinogenesis and synaptogenesis depending on the cell type and area of study [14]. Furthermore, different models of spinogenesis could even coexist in the same cell, reflecting the high degree of plasticity in dendritic spine formation. Three main models of spinogenesis have been proposed: (i) the Sotelo model [25] (ii) the Miller/Peters model [26] and (iii) the filopodial model (see [27], Figure 3).

### 2.1. The Sotelo Model

In 1978, Sotelo proposed that dendritic spines could grow from dendritic trees, independently of the fiber terminals, according to an autonomous cell program of spinogenesis (Figure 3(a)). This model is particularly characteristic for Purkinje cell spinogenesis [14] and it was generated on the basis of morphological data gained from experiments on *weaver* mutant mice that lack granule cells [28–30], *reeler* mutant mice in which granule cell migration is perturbed [31], and neonatal rats irradiated to eliminate the granule cells [32]. The Purkinje cells in these animals bear dendritic spines that have differentiated postsynaptic densities, even though they do not have presynaptic terminals [28–30, 33]. Other experiments have provided data to support this model [34], whereby dendritic spines preferentially form regular linear arrays that trace helical pathways with a short pitch, as suggested earlier [22]. These helical paths have a similar periodicity in fish and mammalian Purkinje cells (both* weaver* and wild-type mice), suggesting that the ordering of the spines around the dendrite is an inherent property of the dendrite to maximize the capacity of the dendritic spines to interact with axons.

**Figure 4:**
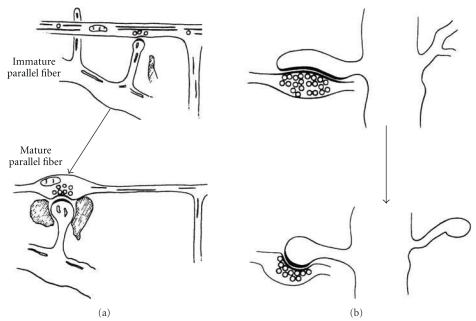
Spinogenesis in the Purkinje cell branchlets contacting parallel fibers according to Larramendi [15, Figures 4 and 5] using electron microscopy, 14-day-old mouse. (a) Dendrites grow “*long spine-like processes,*” once the synaptic contact occurs with a parallel fiber, spines develop a terminal head and the parallel fiber forms a swelling. (b) Parallel fibers often form long synaptic adhesions with developing spines that subsequently decrease in size (*synaptic adhesion waning*).

Nevertheless, in normal animals, Purkinje cell branchlets give rise to long spine-like processes that form synaptic contacts with parallel fibers [15]. Following the onset of these synaptic contacts, the long spine-like processes develops a terminal head, and the parallel fibers form axonal swellings that contain synaptic vesicles. The mature dendritic spine has a big terminal head and a short neck (Figure 4(a)). Cajal [23] described similar long spine-like processes in the Purkinje cell branchlets impregnated by the Golgi method of the 15-day-old cat (Figure 10(b)). Larramendi [15] also found that parallel fibers often form long synaptic adhesions with developing spines (Figure 4(b)). These asymmetric synapses become shorter as the dendritic spine reaches maturity through a phenomenon referred to as “*synaptic adhesion waning*” by Larramendi [15].

*In vitro*, it was found that isolated Purkinje cells had a low rate of survival and they developed a poor dendritic tree covered only with filopodia [35]. However, when Purkinje cells were cultured together with granule cells, they developed a characteristically complex and spiny dendritic tree. A degree of ramification in the dendritic tree might also be necessary to form dendritic spines, and it is noteworthy that when the branchlets of Purkinje cell are not well-developed, dendritic spines were also immature. Moreover, the size of the dendritic spines and their postsynaptic densities appears to be directly related to the activity of the parallel fibers [36]. Indeed, dendritic spines on Purkinje cells undergo changes in morphology and density in association with motor learning [37]. While these alterations mainly affect the dendritic spines of the Purkinje cells connecting with parallel fibers, the population of spines in proximal Purkinje dendritic segments that receive inputs from the climbing fibers (Figure 7(c)) also show different morphological parameters. Although there is a strong genetic regulation of distal spine formation, input activity has an enormous influence on their development and such dendritic spines that contact parallel fibers are smaller and thicker (see [21, 38]; Figure 7(b)) and they are less numerous. In the adult rat, the proximal segment of the Purkinje cell tree has few spines, about 0.01 spines/*μ*m, whereas the spiny branchlets have a density of 0.46 spines/*μ*m [39]. 

Purkinje cells spines that contact climbing fibers appear in clusters of five or six that connect with the varicosity of a climbing fiber (see [22, 40]; Figure 7(d)). These postsynaptic structures are very numerous during development, (as represented in the scientific drawings by Cajal (see [6, 7]; Figure 7(a)) and they are readily observed in his histological slides (Figures 9 (b)–9(d)). However, during development the density of these dendritic spines decreases [21]. Following the chemical destruction of the inferior olive by intraperitoneal injection of 3-acetyl-pyridine [41], the sectioning of olivary fibers [24, 42], or the blockage of nerve activity with tetrodotoxin [36, 38–40, 43] there was an increase in the number of dendritic spines on the proximal segments and soma of the Purkinje cells. Thus, climbing fibers have an inhibitory effect on spinogenesis in the proximal branches and soma of the Purkinje cells, regulating the density of the dendritic spines through activity-dependent mechanisms [38, 39].

### 2.2. The Miller/Peters Model

This model proposes that the dendritic spines of pyramidal neurons directly evolve from synapses on dendritic branches by successive outgrowth of a sessile to a pedunculated appendage (Figure 3(b)). First, synapses are established on the dendritic shafts while in a second step, the presynaptic region of the axon develops a swelling where synaptic vesicles accumulate. Most of these immature dendritic spines are “stubby spines” recognized by their flocculent material. In the third stage, many thin or mushroom-shaped dendritic spines appear and axon terminals show well-developed varicosities [26]. The same sequence of spine formation was observed through electron microscopy in 3, 6, 9, 12, 15, and 21-day-old rat pups and on spiny stellate neurons of the primary visual cortex of one-week-old monkeys (*Macaca nemestrina*; [44]). This model appears to be supported by the large quantity of synapses on dendrites during the initial stages of development [45–50] and the higher number of sessile spines in the initial stages of development when compared to the adult [20, 51]. 

**Figure 5:**
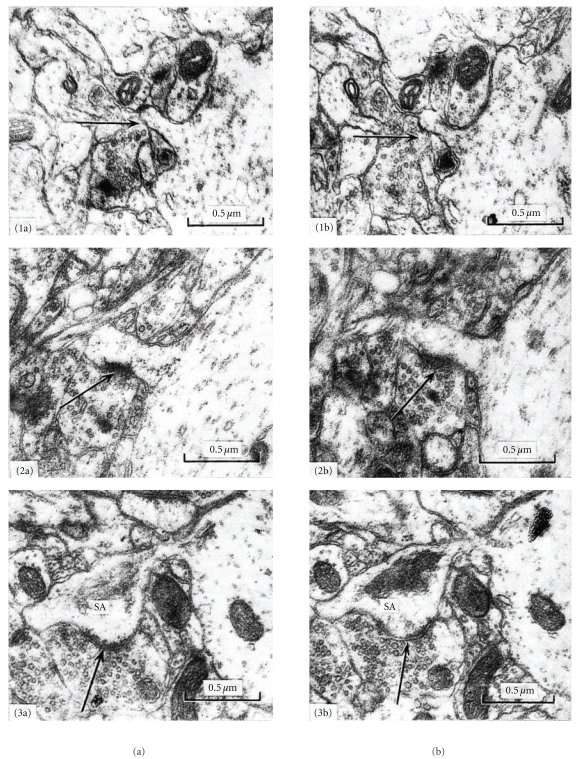
Development of dendritic spines, visual cortex, 19-day-old mouse according to Freire [16, Figures 1, 2, and 3], using serial section electron microscopy. (1a) and (1b) show two medial sections through a dendritic spine in phase 1 of development, the synaptic zone is not developed, but it is contacted by one axon (arrows), no cistern (sacs) of the spine apparatus is present, 19-day-old mouse reared in the dark. (2a) and (2b) show two medial sections through a phase 2 spine, a synaptic zone has appeared (arrows) but is not sacs of the spine apparatus, 19-day-old normal mouse. (3a) and (3b) show two medial sections through a phase 3 dendritic spine with cisterns in its spine apparatus (SA), and a well-developed synaptic zone (arrows).

However, in 19-day-old mice, spinogenesis and synaptogenesis follow a different pattern to that described by Miller and Peters [26] or Mates and Lund [44]. In a quantitative electron microscopy study using serial thin sections, Freire [52] distinguished three phases in the development of dendritic spines in the visual cortex of 19-day-old mice and all 76 of the dendritic spines studied were contacted by at least one axon (Figure 5). In the 1st phase, the volume of the dendritic spines was less than 5 × 10^−2^ *μ*m^3^ (Figures 5, 1(a), 1(b), 2(a), and 2(b)), there were no cisterns in the spine apparatus, and there were no synaptic specializations when the volume was less than 3 × 10^−2^ *μ*m^3^ (Figures 5, 1(a), and 1(b)). In the 2nd phase, the dendritic spines had a volume between 5 × 10^−2^ *μ*m^3^ and 19 × 10^−2^ *μ*m^3^, the spine apparatus had one or two cisterns, and the surface area of their synaptic zones varied between 5 × 10^−2^ *μ*m^2^ and 23 × 10^−2^ *μ*m^2^. In the 3rd phase, the dendritic spines had a volume greater than 19 × 10^−2^ *μ*m^3^, at least three cisterns in the spine apparatus, and a larger surface area of the synaptic zone (greater than 23 × 10^−2^ *μ*m^2^; Figures 5, 3(a), and 3(b)). The linear correlations between the volume of dendritic spines and the surface area of their synaptic zones, and between the volume of the spines and the number of sacs in the spine apparatus, were highly significant (*P* < .01). Therefore, the large variation in size of the dendritic spines in 19-day-old mice might be due to the coexistence of spines at different stages of development. Synapses develop at the same time as the dendritic spines increase their size. Thus, it was suggested that the dendritic spines forms directly from the dendritic shaft when it is contacted by an axon that induces the clustering of Rac1, a small RhoGTPase that pushes the axon and the clustered Rac1 molecules away from the dendrites, forming the spines and recruiting AMPA receptors to synapses [53]. This might provide a mechanistic link between presynaptic and postsynaptic developmental changes. Protracted synaptogenesis was demonstrated after local electrical theta-burst stimulation, by correlating time-lapse two-photon microscopy of newly formed spines on CA1 pyramidal neurons in mice organotypic hippocampal slices with electron microscopy [54]. Among the young spines, only a few hours old (3.2 ± 0.7 h, *n*  =  13), 9 were in contact with axons but did not form synapses, while 4 of those analyzed were not contacted by axons. Indeed, only old spines (16.3 ± 0.4 h, *n*  =  9) form synapses resembling those of the preexisting spines. These old spines had a significantly larger volume than the younger ones and they were indistinguishable from the controls (old, 0.1646 ± 0.0262, *n*  =  9; young, 0.0534 ± 0.0124, *n*  =  13, *P* < .001; control spines, 0.1421 ± 0.0240 *μ*m^3^, *n*  =  14, *P*  =  .55). These data were considered to be evidence that a shaft synapse is not normally converted to a spine synapse by budding from a dendrite [54].

**Figure 6:**
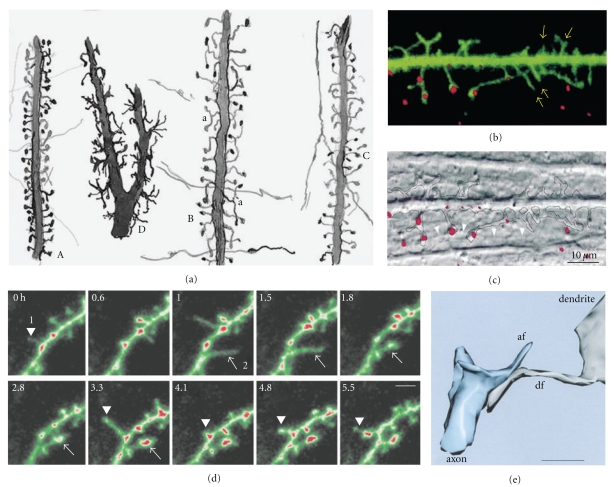
(a) Illustrates a Cajal scientific drawing [17]. The English translation of the original legend of this drawing is in the endnote 3 of the text. We would like to remark filopodia in (D) motor cell, spinal cord, one-month-old cat, and the presence of dendritic appendages with mixed characteristics between filopodia and spines (protospines) in (B) visual cortex, two-month-old child. (b) Synapses at sites of contact between filopodium-like dendritic protrusions and axons [18]. Dendritic appendages of great motility (arrows), a FAST DiO-labeled dendrite (green) and FM4–64-labeled presynaptic buttons (red) in an *in vitro* preparation of 14 days. (c) Differential interference contrast image of the same field. The parallel axon (arrowheads) 5–7 mm below the labeled dendrite (dashed outline) has formed synapses (red) with several dendritic protrusions. (d) New PSD95 clusters emerge in dendritic filopodia that transform into protospines and spines [19]. In a confocal time-lapse sequence of a neuron expressing PSD95-GFP, transient filopodia, devoid of clusters, repeatedly protrude and withdraw at a site (1, 0 h) overlying a shaft cluster. Later, a new filopodium emerges (arrowhead, 3.3 h) and transforms into a cluster-containing protospine (arrowhead, 5.5 h). At another site (2, 1 h), a filopodium extends, persists and stabilizes coincident with *de novo *formation of a cluster (arrow, 1.8–3.3 h), scale bar, 3 *μ*m. (e) synaptic interaction between axonal (af) and dendritic filopodia (df), postnatal day 4, Hippocampus, CA1, three-dimensional reconstruction of electron microscopy images, scale bar 1 *μ*m [20].

Furthermore, time-lapse imaging in live developing hippocampal slices, identified the migration of the PSD (Postsynaptic Density) along the shaft of the dendrites through a dendritic spine or a “protospine” using GFP-tagged PSD-95 (Postsynaptic Density 95) protein (see [19]; Figure 6(b)). However, there was no evidence that PSDs on the dendritic shaft represent a synaptic specialization. Another possible option is that these PSDs could be synthesized at the dendritic shaft and then moved to those dendritic spines or filopodia where synapses are formed. Further support for this model came during dendritic spine loss, following exposure of cortical neuron cultures to NMDA, AMPA, or oxygen and glucose deprivation (similar characteristics of ischemia), the axon terminals remain joined to the swelling formed at the site of the dendritic spine [55]. Two hours after agonist exposure, dendritic spines reemerged in the same location where they disappeared, suggesting that PSDs may contain information for the production of functional dendritic spines. Nevertheless, it should be borne in mind that these pathological conditions might be different from those that might occur during normal development.

The Miller/Peters model does not fit with all the experimental data. Firstly, there are many fewer synapses on dendritic shafts during development than the total number of synapses that exist in the adult neuron, and, hence, sampling during early stages of development might not be representative of the total adult population. It is also possible that prior to the appearance of filopodia and dendritic spines, synapses could only have been established on dendritic shafts. In hippocampal slices, time-lapse imaging studies indicated that dendritic spines may emerge spontaneously from dendrites [19, 27, 54, 56, 57] and also in anesthetized animals [58–60]. Two of these studies focused particularly on the role of the presynaptic terminals. By correlating in vivo imaging studies and electron microscopy, adult CA1 pyramidal neurons in organotypic cultures were seen to give rise to small spines or filopodia after local electrical theta-burst stimulation (TBS) of afferent fibres [54, 60]. These spines initially grow without synapses, and they later formed these functional structures preferentially on preexisting boutons (multisynaptic boutons). Moreover, it was confirmed that in the adult neocortex, the density and the packing of neurites is so high that it might be impossible for axons to first establish synapses on shafts and for them then to move the dendritic spines away, crossing over many other neurites [60]. If this mechanism is active during development, it must be tightly and sequentially regulated so that axons do not block one another. Although synaptic contacts appeared after the emergence of the dendritic spine [54, 60], these studies did not rule out the role of the presynaptic axon in the induction of spinogenesis. Interestingly, preexisting boutons seem to attract the newly formed spines. Since these experiments were performed under TBS condition, nervous activity of the presynaptic terminal could be essential for the dendritic spine formation. Many years ago, several experiments also showed the importance of sensory activity in the development and maintenance of dendritic spines [52, 61–67].

### 2.3. The Filopodial Model

In many neurons, dendritic trees are initially smooth prior to the appearance of dendritic spines and they are only later covered with other types of dendritic appendages called dendritic filopodia (Figure 3(c)). Dendritic filopodia can be distinguished from dendritic spines as they are fine structures (diameter less than 0.3 *μ*m) of 3–40 *μ*m length that do not usually have a bulbous head. Dendritic filopodia are much more dynamic than dendritic spines [27], and in hippocampal pyramidal neurons, most of the dendritic filopodia continuously extend from and retract back to the shaft of the dendrite with a half-life of about ten minutes [18, 27]. The existence of dendritic filopodia during development has often been described [18, 19, 24, 26, 27, 68–74], and their morphological similarity coupled with the sequential appearance of dendritic filopodia and spines suggest that filopodia might be precursors of dendritic spines. Accordingly, the “filopodial model” proposes that filopodia differentiate into dendritic spines following their interaction with axons (Figure 3(c)). By contrast, electron microscopy studies have shown that there are many synapses on dendritic shafts and at the base of filopodia, suggesting that filopodia may be particularly implicated in synaptogenesis by recruiting synapses to the shafts [20]. Thus, dendritic filopodia would retract and incorporate the synapses into the dendritic tree, and, only later, the dendritic spines grow at the sites at which these synapses are found (in a manner similar to that of Miller/Peters model). Studies on postembryonic dendritic remodelling of motoneuron 5 during the metamorphosis of the hawkmoth *Manduca sexta* [75], indicated that presynaptic terminals readily accumulate along the filopodia towards the dendrites.

In recent years, research has focused on dendritic filopodia to shed light on the function of these structures. Ultrastructural analyses have demonstrated their implication in synaptogenesis whereas *in vivo* time-lapse imaging has defined the dynamics of these structures. However, long ago scientists had already proposed their existence, motility and probable implication in synaptogenesis. It is noteworthy that Cajal referred to similar developmental structures many years ago, and although they appeared in his drawings (Figure 6(a)), he did not specifically name them. Cajal proposed that they were transient structures, as later confirmed, and in reference to Figure 6(a), Cajal wrote:

*“…mostramos en la figura 50 algunos dibujos tomados de tallos de pirámides cerebrales adultas o jóvenes. En A presentamos el tallo de una pirámide de la región visual del conejo casi adulto. Nótense cuán cortas son las espinas y cómo empiezan delgadas y acaban por un bulbo final. Son pocas las bifurcadas. En B hemos dibujado otro tallo del niño de dos meses (pirámide de la región visual). Llama la atención, no sólo la mayor longitud de los apéndices, sino su frecuencia con que se dividen y los cambios de dirección de sus ramillas secundarias. En C copiamos un tallo de las pirámides del gato de un mes. Confírmase la disposición mostrada en A; las espinas aparecen un poco más largas y con frecuencia incurvadas. Como término de comparación hemos dibujado en D una dendrita de una célula motriz de la médula (gato de un mes). Adviértase que la superficie está erizada de proyecciones irregulares y rara vez acabadas mediante bulbos. Es casi seguro que esta disposición es transitoria. En fin, dibujamos también algunas colaterales nerviosas cruciales u oblicuas (cerebro), sin que sea dable apreciar su fusión con las espinas” *(Cajal, 1933) [17]. ^3^

Previously, Cajal had also described filopodia in growth cones:

*“En la porción terminal ó base, no es raro ver una prolongación membranosa más larga, especie de avanzada protoplásmica insinuada en los intersticios intercelular ó interepitelial” *(Cajal, 1899) [6, page 514]. ^4^

The filopodial motility was suspected by several scientists, including Morest, who even proposed a role for these structures in synaptogenesis:

*“...they help to pull the afferent axonal end-branches to their definitive synaptic location.”* (Morest, 1969a, b) [68, 69].

Filopodia motility was also demonstrated in growth cones [76], although the motility of dendritic filopodia was demonstrated by Dailey and Smith in 1996 [27], who found that:

*“...most of the filopodial protrusions (up to 10* *μm long) extended (maximal rate: 2.5* *μm/min) from dendrite shafts, then retracted back to the shaft within 30 minutes or less (median lifetime, 10 min).” *

In addition to the great abundance of dendritic filopodia during active synaptogenesis [24, 26, 68, 69, 73, 77], their elongated morphology suggests that these structures could play a role as bridging structures to facilitate axodendritic synaptic contacts [18, 26, 27, 68, 69, 74, 78–83]. Moreover, filopodia could act either passively to form a virtual dendrite or actively to define the dendritic capture volume [18, 27]. The implication of filopodia in synaptogenesis has been elucidated by electron microscopy studies that identified synapses in both dendritic growth cone filopodia [78, 81, 83, 84] and collateral dendritic filopodia in different areas of the nervous system (see [20, 74, 85], Figure 6(e)). Interestingly, high-frequency focal synaptic stimulation induced enhanced growth of small filopodia-like protrusions in CA1 hippocampal dendrites of organotypic slices prepared from rat pups at postnatal day 7 and cultured for 7 to 9 days. This process is stimulated by synaptic transmission mediated by activation of NMDA receptors, since the presence of APV (DL-2-amino-5-phosphonovaleric acid), a specific antagonist for NMDA receptors, abolished the growth of filopodia. The synapses in these filopodia are more likely to connect with active presynaptic axons during the synaptic stimulus, providing a mechanism to explain Hebbian rules of synaptic plasticity [86]. Overexpression of GluR2 receptors (the glutamate receptor 2 subunit of AMPA receptors) increases the length of spines, the width of spine heads, and the density of spines in mature cultured hippocampal neurons (22 days *in vitro*) that normally exhibit mushroom-like spines. However, in younger neurons (11 days *in vitro*), GluR2 overexpression only induces an increase in the density of filopodia-like protrusions that were longer and wider than those found on control neurons (see [87]). The first N-terminal 92 amino acids of the extracellular domain of GluR2 are necessary and sufficient to promote the appearance of spines through the formation of a synaptic complex with N-cadherin [88]. In addition, during the development of the rat barrel cortex *in vivo*, sensory deprivation by whisker trimming markedly reduces the protrusive motility of filopodia and dendritic spines in layer 2/3 pyramidal neurons of deprived regions during a critical period around postnatal days (P) 11/13, but no effect was observed in either younger or older animals. The density, length, or shape of spines and filopodia did not change. Thus, it was concluded that sensory deprivation does not modulate synapse number itself, but, rather, it modifies the experience-dependent rearrangements of synaptic connections required to form precise sensory maps, as shown by the electrophysiological study of layer 2/3 receptive fields [89].

In addition to GluR2 receptors, overexpression [90] or clustering [91] of TM-agrin (a transmembrane form of the extracellular matrix heparan sulfate proteoglycan agrin) induces the formation of highly dynamic filopodia by the activation of intracellular signalling cascades (involving lipid rafts, Src family kinase Fyn, MAPK), supporting the hypothesis that TM-agrin is a receptor or coreceptor in neurons [92]. Indeed, activity-dependent presynaptic exocytosis of neurotrypsin and the resulting proteolytic cleavage of agrin are a mechanism to promote the activity-dependent formation of dendritic filopodia [93]. The cleavage of agrin requires the additional activation of the postsynaptic cell, and, thus, neurotrypsin-dependent cleavage of agrin could represent a molecular detector for concomitant presynaptic and postsynaptic activation. The activation of SK3 channels (small conductance Ca^2+^ activated K^+^) also leads to immediate filopodial sprouting and the translocation of the protein into these novel filopodial protrusions in neural progenitor cells [94].

Diminished calcium transients in dendrites facilitate filopodial outgrowth during development of organotypic slices of the neonatal rat hippocampus (P 0–2 + DIV 1–3). Filopodia can generate calcium transients that probably lead to immature synapse formation when transmitted into the dendrite, preventing the growth of additional filopodia in a range of 10 *μ*m, thereby impeding the overload of synaptic inputs on some dendrites as well as underrepresentation of inputs on others [95]. Global calcium activity in the hippocampus is dependent on GABA signalling at these ages. However, since local calcium transients are not completely abolished by blocking GABA receptors, other signalling molecules must also be involved in inducing local calcium transients, such as receptors that recognize adhesion molecules and extracellular matrix components like *β*1 integrin or neurotrophins [95].

In addition to the similar morphology and sequential appearance of dendritic spines and filopodia [26, 51, 96, 97], other studies also suggest that dendritic filopodia evolve to form the definitive dendritic spines [18, 27]. On the one hand, there was a progressive decrease of dendritic filopodia and an increase in dendritic spines during development, suggesting that filopodia could transform into dendritic spines [27]. In addition, an intermediate transient stage was detected, the *protospine*, with the dynamic characteristics of both dendritic spines and filopodia. Protospines were stable for up to 22 hrs (the half-life of filopodia is only 10 minutes), but they had the motility of filopodia. Dendritic filopodia were also seen to interact with synaptic boutons 5–10 *μ*m away from the dendrite, and the dendritic filopodia that establish synaptic contacts were more stable and less motile with a differentiated head (Figure 6(b); [18]). Some dendritic filopodia have a swelling on their synaptic contacts [20, 98], and, thus, a bulbous head appears to be a characteristic of dendritic spines and protospines that promote the stabilization of the filopodia.

Dendritic spines with several heads, *multispines*, were described in Golgi-impregnated or Lucifer Yellow-filled neurons of the medial hyperstriatum ventrale of 3-day-old chicks [99], a brain area implicated in learning and memory processes in the chick [100]. The multispine pedicle or neck can be branched or unbranched, and the number of heads per multispine ranged from 2 to 8 (mean = 3) for branched multispines, and from 2 to 4 (mean = 2) for unbranched multispines. Multispines represented 16.4% of the total number of spines, but if the number of spine heads is considered, this percentage increases to 34.69%. Multispines are also found in all neuronal nuclei studied in 3-day-old chicks, for example, the Ectostriatum, field L, hyperstriatum ventrale, lobus parolfactorius, and neostriatum. 

These *multispines* [99] are similar structures to the *protospines* [27], and they were previously considered as a transitory phase in the development of dendritic spines. For instance, Cajal drew dendritic appendages with intermediate characteristics between filopodia and spines in the visual cortex of a 2-month-old child (Figures 6(a) and 6(b)). In a trisomic infant, Marín-Padilla [101] considered the “long (hair-like)” dendritic spines with several “dilatations in their pedicles” as retaining “their primitive developing appearance,” suggesting that inadequate “en passant” contacts formed because the spines failed to develop mature spines (shorter and one-headed spines). Multispines or protospines can also be seen in his pictures and drawings of neurons in normal developing cerebral cortex (see Figures 2(c) and 2(e) in [102]).

We have found long appendages similar to the protospines, with one or more heads and morphological characteristics between dendritic spines and filopodia, in Cajal's histological slides (Figures 10(c), 13(d), 14, 15, 16, 17, 18, and 19). Interestingly, *de novo* synthesis of GFP-tagged PSD-95 may occur in filopodia (Figure 6(d)) that sometimes develop shapes similar to those we have seen in Cajal's histological slides, exhibiting characteristics of both dendritic spines and filopodia [19].

However, there is no clear evidence that dendritic filopodia are the precursors of all dendritic spines. Although Dailey and Smith [27] occasionally observed the apparent conversion of an actively protrusive dendritic filopodium to a more stable spine-like structure, they could not correlate this conversion with functional synapse formation in their slice preparation. Using two-photon time-lapse imaging of developing hippocampal pyramidal neurons, it was confirmed that most filopodia turn into sessile and pedunculated spines, although filopodia could also extend out of existing spines [57]. De Roo et al. [103] established that filopodia only occasionally led to the formation of durable dendritic spines in a more general process that stabilized new protrusions, as determined by the enlargement of the spine head and the expression of PSD-95 within a critical 24 h period. PSD-95 controls synapse formation by regulating nNOS (neuronal nitric oxide synthase) expression at the synapse, releasing NO (nitric oxide), and activating cyclic guanosine monophosphate (cGMP) signalling in presynaptic axons. Multi-innervation (up to seven presynaptic terminals) of large dendritic spines occurs when PSD-95 is overexpressed in rat hippocampal slice cultures maintained for 11–15 days *in vitro *[104].

There is also other data suggesting that filopodia are probably not the precursors of all dendritic spines. Some studies indicated that dendritic filopodia disappear completely before the appearance of the dendritic spines [20, 68, 69]. Moreover, many neurons that do not have dendritic spines in adulthood bear dendritic filopodia during the synaptogenic period [24, 70, 73, 77, 105–107]. However, although we have observed dendritic filopodia in double bouquet cells and basket cells of the cerebral cortex and on basket cells and Golgi cells of the cerebellar cortex, the number of filopodia is very low when compared with the number of filopodia of pyramidal cells in the cerebral cortex. In contrast to the filopodial model, some experiments show that dendritic spines could develop *de novo* from dendritic branches [27, 57]. However, these experiments do not rule out the possibility that spines are born *de novo* without synapses, as demonstrated in adult animals [60] in which spines emerge as thin dendritic spines or as short filopodia that later appear to form a differentiated head. Similarly, nonsynaptic dendritic spines in the neocortex that resemble dendritic filopodia have been seen [108], although smaller than those observed during development, suggesting that they could be new spines formed in adult animals. It is possible that adult neurons show smaller dendritic protrusions than the dendritic filopodia observed during development, possibly because during development there is more free space in the neuropil and the axons are further apart (see supplementary video 1 available online at doi: 10.1155/2011/769207).

Besides participating in synaptogenesis and spinogenesis, dendritic filopodia are also implicated in the selection between presynaptic candidates when forming the correct nervous connections. Thus, dendritic filopodia can select synaptic partners between axons (excitatory versus inhibitory: [109]). Indeed, when filopodial contacts are stabilized with excitatory terminals, high-frequency local calcium transients independent of glutamate are evident whereas short-lived contacts, especially with inhibitory terminals, are associated with low-frequency local calcium transients.

Dendritic filopodia might also be involved in the specific branching of the dendritic tree. Filopodia can be formed on the terminal dendritic growth cones, and according to the synaptotropic hypothesis [83], filopodia could receive synaptic contacts forming a dendritic branch holding the synaptic contacts. By successive iteration, the definitive adult dendritic tree would be formed. Two kinds of filopodia might exist, each with different properties and functions [110]: collateral filopodia and dendritic growth cones filopodia. Filopodia of the dendritic growth cones are involved in dendrite growth and branching in an activity independent manner whereas shaft filopodia are responsible for activity-dependent synaptogenesis, and, in some cases, they may become dendritic spines. Two similar types of filopodia were also found in the hawkmoth *Manduca sexta* [75].

## 3. Observations from the Histological Slides and Original Scientific Drawings of Ramón y Cajal and Their Correlation with Recent Research

The Cajal Museum holds 4529 histological slides personally prepared by Santiago Ramón y Cajal. Of these, 809 are stained by the Golgi method, which enables us to visualize splendidly preserved dendritic spines and filopodia. Although there are 109 histological slides stained with the methylene blue method, which also enables filopodia and dendritic spines to be observed, these preparations were not used in this study due to their poor preservation. 

Here, we have employed the following methods.

**The Age of the Animals**

The age of the animals had to be determined by the labeling of Cajal's histological slide [111]. Sometimes, Cajal did not write the exact prenatal and postnatal day of development of animal, he used the month or phrases indicating the stage of development like “*almost newborn*,” “*newborn rabbit*,” and “*almost adult*.” We have found that this was sufficient for the type of analysis carried out in this work.

**Focus Extended Imaging**

We have obtained stacks of digital images of the histological slides and the areas out of focus in each image were eliminated using *Image J* software.

**Three-Dimensional Reconstructions and Rendering**

We used the *Neuronal Coding, Neuronal Quantification, and Neuronal Transformation* programs [16, 112, 113] for the 3D-reconstruction and quantification of neuronal structures (dendrites, axon, dendritic spines, dendritic filopodia, and varicosities). We also developed a computer program to convert the codes used in the *neuronal programs* to the *POV-Ray* (persistence of vision raytracer: http://www.povray.org/), a freely available program that we used for rendering.

### 3.1. Cerebellum: The Purkinje Cell

The Purkinje cell experiences different phases of development characterized by their dendritic appendages. We have studied developmental stages III to VI since we were unable to find cells in the two first stages in the Golgi-impregnated histological slides. Cajal [114] described the first or fusiform phase using the reduced silver nitrate method, but as it does not stain dendritic appendages, we did not study any of the histological preparations stained in this way. In addition, an intermediate phase (*phase of regressive atrophic dendrites*) between the *fusiform phase* (I) and the *stellate cell phase with disoriented dendrites *(III) has also been described [115–117]. Thus, the successive phases of the development of the Purkinje cell are listed below.

**(I) Fusiform Stage**

During this phase, the dendrites extending from both sides of the soma are smooth, and they do not have any appendages. This stage of development extends from 12 to 14 days of incubation in the chicken according to Cajal.

**(II) Regressive-Atrophic Dendrite Phase.**

There is an intense regressive process with the reabsorption or retraction of the long dendrites. The Purkinje cell becomes reduced to the soma with no branches or with short processes.

**(III) Dendritic Disorientation Stage**

This stage is characterized by the explosive outgrowth of short perisomatic protrusions emerging in all directions. We observed some cells with this morphology in histological preparations from a newborn dog, and the dendrites evidently do not develop spines (Figures 8(a)–8(g)), although they have both lateral (Figure 8(b)) and terminal dendritic filopodia, the latter being especially notable (Figures 8(c) and 8(d)).

**(IV) Dendritic Regularization Stage**

The size of the soma increases to 15–20 *μ*m, and the Purkinje cells have a differentiated apical trunk with secondary and tertiary branches (Figure 9(a)). Basal dendritic branches also emerge from the soma, but they are usually shorter than the apical ones. Both the basal and apical branches bear dendritic spines (Figures 9(b)–9(d)), and dendritic filopodia are frequently seen along the dendritic branches and at their tips (Figures 9(g) and 9(h)). The branches with filopodia are typically observed at the base of the soma, near the emerging axon (Figures 9(e)–9(g)). Cajal did not identify dendritic spines or filopodia at this developmental stage, although both spines and filopodia are carefully represented in his scientific drawings (Figures 9(b)–9(g)). It is interesting to note that the perisomatic branches also develop dendritic spines, although these will later be reabsorbed.

**(V) Disappearance of Perisomatic Branches, Emergence of Secondary and Tertiary Branches on the Apical Trunk, and Appearance of Dendritic Spines**

As development progresses, the dendritic tree increases in size (Figures 10–12) engendering a single apical trunk with higher-order branches. The branchlets (small terminal branches) and the dendritic spines contact the parallel fibers. We have found different neuronal types in this phase of development, the more immature of which were observed by Cajal in newborn cats (Figure 10(b)). These neurons have dendritic spines (Figure 10(a)2-3), especially on the proximal primary branches whereas the high-order branches bear several thin dendritic spines (Figure 10(a)2). There are numerous filopodia located both laterally and on the terminal portions of the dendritic branches (Figures 10(a)1, 10(b), and 10(c)). In 17-day-old dogs (Figures 11(a)–11(d)) and in young “*almost adult*” cats^5^ (Figures 11(e)–11(h)), the ramifications and density of the dendritic appendages in the dendritic tree increase, and the filopodia are predominantly located at the terminal portion of the branchlets (Figures 11(b)–11(f)). Some collateral filopodia can be found in the Purkinje cells of 17-day-old dogs (Figures 11(b) and 11(d)2) and their branchlets may also bear thin dendritic spines (Figures 11(c)2 and 11(d)3). There were many sessile dendritic spines or spines with no clear differentiation between the head and neck that decrease in Purkinje cells of the young “*almost adult*” cats^5^ (Figures 11(f), 11(g)2-3, and 11(h)3-4). Furthermore, there were some thorny structures (Figure 11(c)4) that were defined as “*budding branchlets*” [118].

In contrast to the cat and dog, the 12-day-old mouse has more types of dendritic spines (i.e., sessile, thin, mushroom, and ramified) that are more mature than in a cat or a dog of a similar age (data not shown).

**(VI) Adult Stage**

We found three histological slides prepared by Cajal of the translobular cerebellum from adult animals (cat, bird, and man) impregnated by the Golgi method. The dendritic tree of the Purkinje cell is completely developed, although the degree of ramification varies with the species (Figures 12(a) and 12(b)), and in birds, Purkinje cells are less ramified than those of the cat or human. In the adult phase, the dendritic trees are densely covered with different types of dendritic spines (thin, ramified, mushroom, sessile: Figures 12(c)–12(e), 12(i)–12(n)), but there is also a significant difference between the proportion of each type in each species (Figures 12(f)–12(h)). Thus, the Purkinje cell tree in birds has more sessile dendritic spines than in mammals indicating a progressive differentiation of the neck and the head of the dendritic spine along the phylogenetic scale.

#### 3.1.1. Conclusions Concerning Spinogenesis in Purkinje Cells of the Cerebellum

We will present our conclusions on the development of Purkinje cells dendritic spines under three headings in relation to the three known types of dendritic appendages: dendritic filopodia, dendritic spines contacting climbing fibers, and dendritic spines contacting parallel fibers.

**(1) Dendritic Filopodia**

We observed dendritic filopodia associated with Purkinje cells at developmental stages (III), (IV), and (V) (Figures 8–10), and the density of filopodia along the dendritic tree of the Purkinje cell changed according to these developmental stages. Filopodia found at the tip and the terminal portion of the dendrites (Figures 8(c), 8(d), and 8(f)), and at the lateral branches of the definitive apical trunk (Figure 9(h)) are probably implicated in the orientation and growing of the dendrites. While filopodia on branches that emerge laterally from the soma (Figures 9(e) and 9(f)) are probably implicated in the interaction with the climbing fibers at this developmental stage.

Later on, in the Purkinje cells, we found dendritic appendages with morphological characteristics of both dendritic filopodia and spines (Figures 11(b), 11(c)1 and 11(d)1), like the *protospines *in hippocampal tissue slices [27]. Protospines have a gelatinous appearance, they are stained light brown by the Golgi method, and they contain one or more bulbous heads. Previously, the collateral filopodia and protospines were also observed in the cerebellum of 5, 10, and 15-day-old rats whereas they are rare in 30-day-old rats (Figure 10(d): [24]). The presence of these dendritic appendages suggests that synaptogenesis and spinogenesis can be also mediated by filopodia in the Purkinje cell. Interestingly, Cajal observed similar collateral filopodia in his drawings (Figure 10(b)), and describing the Purkinje cell dendrites in a 15-day-old cat, he wrote:

“*Casi todas sus ramas *(*Figure  2(b), Figure  163 de este trabajo*)* poseen ligeras espinas perpendi-cularmente insertas en su contorno. Estas espinas aparecen teñidas en café claro y son más grandes que las de las ramitas terminales de los corpúsculos adultos*” ^6^ (Cajal, 1889) [119].

**(2) Dendritic Spines Contacting Climbing Fibers**

**Figure 7:**
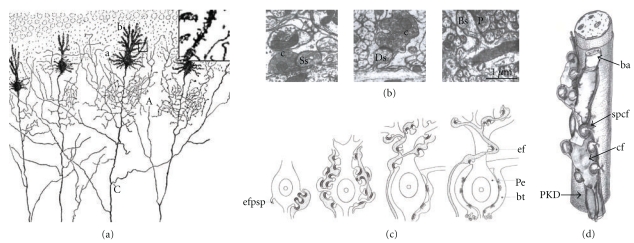
(a) Illustrates a scientific drawing by Cajal [7, Figure 418]. Purkinje cells, cerebellum, newborn child, and Golgi method. Supernumerary collaterals of the Purkinje axon (A); inferior collaterals (C); soma with indifferent appendages (a); formation of the definitive branches (b); inset shows dendritic spines digitally enlarged of the boxed region. (b) Purkinje cell; from left to right, perisomatic spines synapsing (Ss) on climbing fibers (c); dendritic spines (Ds) of large dendrites synapsing on climbing fibers (c); dendritic spines of Purkinje cell dendritic branchlets that synapses on parallel fibers (p; [21]). (c) sketch of the evolution of the climbing fibers and dendritic spines based on electron microscopy observations [15]. (d) cluster of dendritic spines (spcf) connecting with a climbing fiber (cf) [22].

**Figure 8:**
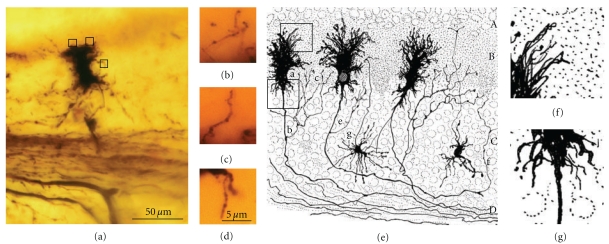
(a) Shows a Purkinje cell in the phase of dendritic disorientation, cerebellum, new-born dog. Cajal histological preparation, Golgi method. Boxed regions show collateral filopodia (b) and terminal ones (c, d). (e) Cajal scientific drawing [7, Figure 416]. Purkinje cells, several days-old dog. Boxed areas (f, g) show digitally enlarged filopodia.

**Figure 9:**
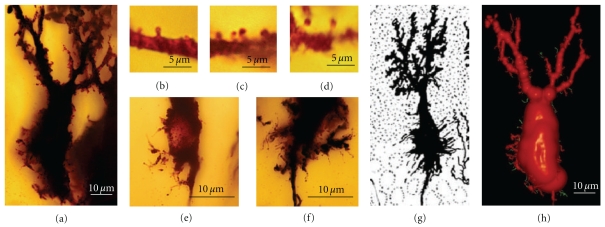
(a) Represents a Purkinje cell in the phase of dendritic regularization, cerebellum, new-born dog. Cajal histological preparation, Golgi method. (b, c) Dendritic spines of apical branches. (d) dendritic spines of lateral perisomatic branches. (e, f) Purkinje cells with perisomatic branches with filopodia near the axonal origin. (g) Cajal scientific drawing [7, Figure 417]. Purkinje cell, several days old dog. (h) Three-dimensional reconstruction of the Purkinje cell showed in (a); filopodia are color coded in green.

We observed dendritic spines on the lateral dendrites of the soma (Figure 9(d)). According to Larramendi and Victor [21] and Laxson and King [120], these dendritic spines receive synaptic contacts (Gray type I) from the climbing fibers. Later on in development, the perisomatic primary dendrites will be reabsorbed, and only perisomatic dendritic spines will remain, receiving connections from climbing fibers (see [21, 120]; Figures 7(b)–7(d)). In fact, we did not observe these dendritic spines in adult animals, although a small population should still be present.

**(3) Dendritic Spines Contacting Parallel Fibers**

**Figure 10:**
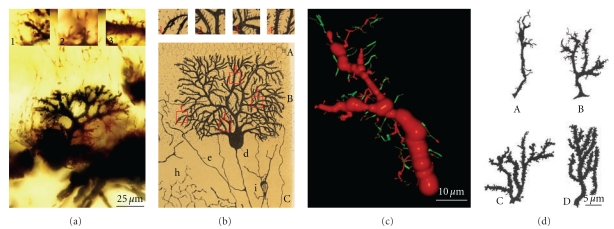
(a) Shows Purkinje cell forming the dendritic branchlets, Cajal histological preparation, Golgi method, newborn cat. Insets: 1, terminal filopodia; 2, thin dendritic spines; and 3, dendritic spines contacting climbing fibers. (b) Purkinje cell, 15-day-old cat. Insets show filopodia and thin dendritic spines, Cajal scientific drawing [23, Figure 2]. (c) Purkinje cell, apical branch, three-dimensional reconstruction showing dendritic filopodia and protospines (green) and few dendritic spines (red), newborn cat. (d) Purkinje cell, dendritic filopodia, protospines and dendritic spines, rat of 5 days (A), 10 days (B), 15 days (C), and 30 days (D); scientific drawing of Berry and Bradley [24].

**Figure 11:**
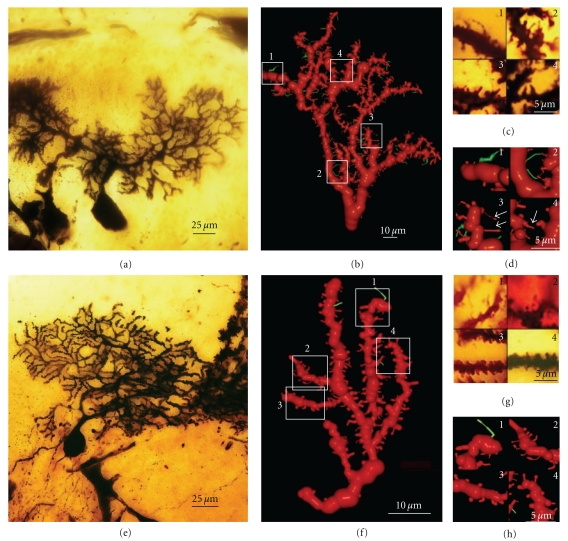
(a) Illustrates a Purkinje cell, 17-day-old dog, Golgi method, Cajal histological preparation. (b) Three-dimensional reconstruction of a portion of the dendritic tree of this Purkinje cell. (c) Golgi-impregnated dendritic appendages of this Purkinje cell; 1: protospine; 2: thin dendritic spines; 3: small dendritic spines; 4: possible budding branchlets. (d) Boxed regions in (b); 1: protospine (green); 2: thin filopodia (green); 3: thin dendritic spines (arrow); 4: possible budding branchlet (arrow). (e) Purkinje cell, “cat almost adult” according to the label of the Cajal histological preparation, Golgi method. (f) Three-dimensional reconstruction of a portion of the dendritic tree of this Purkinje cell. (g) Golgi-impregnated dendritic appendages of this Purkinje cell; 1, dendritic filopodia; 2, thin dendritic spines; 3 and 4 sessile dendritic spines. (h):Boxed regions in (f); 1, dendritic filopodia; 2, thin dendritic spines; 3, 4, sessile dendritic spines and some thin dendritic spines.

The greater density of sessile dendritic spines on Purkinje cell branchlets of “cat almost adult” (Figures 11(f), 11(g)2, 3, and 11(h)3, 4) suggests that the majority of dendritic spines could emerge from dendrites through progressive growth, and only later will many dendritic spines differentiate their head and neck, becoming pedunculated spines. However, there are many collateral filopodia and protospines in newborn cats (Figure 10(c)) that could evolve directly into dendritic spines.

### 3.2. Cerebral Cortex: Pyramidal Cells

Pyramidal cells follow a stereotypic developmental program, and the dendritic appendages initially described by Cajal [6, 7] serve to characterize the different stages of development. However, there are two phases that Cajal described with the reduced silver nitrate method that we have been unable to study, as this procedure does not impregnate neuronal appendages: the primitive bipolar stage and the neuroblast stage. Thus, our observations are limited to the subsequent phases.

**(I) Primitive Bipolar Phase**

Cajal studied this stage through the reduced silver nitrate method in the cerebral rostral vesicle of 3–4-day-old chicken embryos. Two opposite processes emerge from the soma, one directed towards the cerebral vesicle surface whereas the other is directed towards the inner side.

**(II) Neuroblast Phase.**

The peripheral process disappears whereas the inner one remains and develops into an axon with a growth cone at its tip. This axon can only be observed during the initial stages of development, as it is very difficult to detect in the fetus and newborn animals.

**(III) Bipolar Phase (Figures 13(a) and 13(b)).**

The first outgrowth from the soma forms the axon, which is followed by the emission of an apical dendrite that reaches layer I, giving the neuron its *bipolar shape*. During this stage, the neurons appear to have a smooth surface with no dendritic appendages. However, in some cells, small appendages emerge from the soma and the apical trunk (Figure 13(b)). Some bipolar cells also contain a basilar branch that usually ends in a ball.

**(IV) Phase of Basilar Dendrite Appearance and of Collateral Oblique Dendrites of the Apical Trunk**

The pyramidal cells emit basal dendrites, oblique branches of the apical trunk (initially those more proximal to the soma), and the apical tuft, as observed in Cajal's histological preparations of the newborn rabbit (Figure 13(c)) and human fetus (Figure 13(e)). During this stage, the dendrites usually end in a ball or in a dendritic growth cone. The dendritic branches are smooth or with some appendages (filopodia, protospines, and thin and mushroom spines; Figure 13(d)1–8).

**(V) Phase in Which Nervous [Axonal] Collaterals Appear**

These collaterals first appear on big pyramidal cells and during the following days, the small pyramidal cells also develop axon collaterals. During this phase, the dendrites (apical tuft, Figures 14(a) and 14(b); apical trunk, Figures 14(c)–14(e); basilar dendrites, Figures 14(f) and 14(g); oblique apical dendrites, Figure 14(h); see also Figures 15(a)–15(d) and 16(a)–16(c), supplementary video 2) develop many dendritic filopodia, protospines, and some dendritic spines. Later, the ratio is reversed with many more dendritic spines than dendritic filopodia and protospines (Figures 15, 16(d)–16(f), and 17(e), supplementary video 3). In Cajal's histological slides, we were able to see different forms simultaneously (dendritic spines, protospines, and filopodia), although there were successive periods when dendritic filopodia and protospines or dendritic spines were very abundant, and the evolution was gradual. Moreover, spinogenesis did not occur simultaneously in each neuron, and some dendritic branches developed spines earlier (Figure 17(e)) than others (Figure 17(d)). We also found that dendritic spines and filopodia coexist, especially in neurons of newborn animals and in the first few day of life. In addition, there are many protospines more than 5 *μ*m long, sometimes ramified with terminal bulbous heads (Figures 14, 15, 16(b), and 17) and/or with heads along the protospine stalk (Figures 14, 15(b), and 16(c)). These kinds of protospines were also visible in later stages of development.

The density of dendritic appendages increased during development, although the proportion of dendritic filopodia fell. We found more dendritic spines in more mature stages, such as Cajal's histological slides shown in Figures 17(d) and 17(e). Interestingly, we also found many dendritic spines emerging from the dendritic tree that did not have a clear distinction between head and neck. For instance, the distinct proportions of the dendritic appendages were 64.7% thin spine, 16.8% sessile spine, 14.7% mushroom spine, 2.41% branched spine, and 1.39% dendritic filopodia, in a young mouse pyramidal cell visualized in a histological preparation of Cajal [2]. These sessile spines are probably dendritic spines emerging *de novo* from the dendritic branches.

#### 3.2.1. Conclusions about Spinogenesis in Pyramidal Cells of the Cerebral Cortex

Dendritic appendages only emerge when basilar dendrites appear (Figure 13(b)). Such appendages could be filopodia sensing the local environment in order to initiate the formation of basilar branches in the correct position and orientation. We found few dendritic spines during fetal periods and in newborn animals (Figure 13(d)) despite the presence of some dendritic filopodia. In successive developmental stages, dendritic appendages increase in number, and our observations indicate an initial proliferation of dendritic filopodia. Although, some studies suggest that the dendritic filopodia completely disappear before the appearance of dendritic spines [20, 68, 69], we observed filopodia and dendritic spines coexisting in many different neurons. The progressive decrease in filopodia and the progressive increase in dendritic spines could suggest that filopodia give rise to dendritic spines. It is noteworthy that we found many appendages with a mixed morphology between dendritic spines and filopodia (protospines). Protospines could either evolve into dendritic spines (filopodial model) or retract to the dendritic shaft [20], and thus, the formation of dendritic spines could follow the Miller/Peters model.

However, dendritic spines also appear to emerge directly from the dendritic shafts, especially in advanced stages of development (Figure 15(c)). During adulthood, owing to the reduction of the extracellular space, dendritic spines could emerge from the dendritic shafts as short filopodia or as spines with no synapses to form the definitive dendritic spines. Thus, small dendritic protrusions with no clear difference between the head and neck were described in, the adult as resembling the structures we found during development [108]. Also in the adult, the emergence of *de novo* dendritic spines from dendritic trunks was also described to only later develop a synapse [60].

### 3.3. Olfactory Bulb: Granule Cells

We have studied eleven Golgi-impregnated histological slides of the olfactory bulb of newborn and one-month-old dog, and mice. We only found granule cells in the developmental stage when the dendritic tree is completely formed, and these granule cells have a peripheral dendrite that ends in a dendritic tuft and a number of short inner dendritic branches (Figure 18(a); supplementary video 4). The basilar and apical branches of these granular cells have many long filopodia and protospines (mean size = 5.77 *μ*m) that are frequently ramified (Figure 18(b)). These branches also bear different types of dendritic spines, especially thin and mushroom spines. Like the pyramidal cells, there are many filopodia at the primary stages of development, while later, the granule cells become covered by protospines and dendritic spines (Figure 18(b)). Sometimes, these protospines have more than one varicosity (Figures 18(b)-16 and 18(b)-18). Interestingly, there are also some dark-brown dendritic appendages that differ from the light-brown Golgi-impregnated dendritic filopodia found on the apical tuft. These also have morphological characteristics between those of filopodia and dendritic spines, with very long necks and big heads. However, based on the dark color of the Golgi staining, we think that they are more stable structures than the protospines and that they could be the *gemmules *described by Rall and Shepherd [121] in the adult dendritic tree of the granule cell (Figures 18(b).18, arrow). The soma of the granule cell also has thick and slim appendages. Sometimes, the thick appendages also cover the beginning of the dendritic branches. A 3-D reconstruction of a granule cell from a one-month-old dog can be seen in the supplementary video 4.

#### 3.3.1. Conclusions about Spinogenesis in the Granule Cells of the Olfactory Bulb

Granule cells of the olfactory bulb are characterized by their small size and the absence of axon [122]. Cajal confirmed this discovery of Golgi and described the connection of these granule cells with mitral cells and the existence of dendritic spines on the granule cell branches:

“*...la expansión periférica de los granos posee una orientación y conexión invariables, toda vez que se dirige constantemente á la zona plexiforme, donde se termina á favor de un penacho de ramas fuertemente espinosas, en contacto con las dendritas secundarias nacidas en las células mitrales*” (Cajal, 1904) [7, Page 927]. ^7^

Filopodia are very common during the initial steps of development, protospines are especially common during the next phases whereas dendritic spines are commonly found in the histological preparations of more mature animals. These protospines could interact with different presynaptic partners in a kind of synaptogenic competition, as supported by the confirmation of filopodia as multisynaptic structures [20, 123]. Besides the dendritic filopodia and protospines, long dendritic spines with a big head (*gemmules*) can be observed in the dendritic tuft of granule cell in the olfactory bulb [121]. These dendritic spines were 5 to 6 *μ*m in length, with a head of 1–2 microns [124]. Electron microscopy [125–127] confirmed that gemmules are both inhibitory presynaptic and excitatory postsynaptic elements [128], and reciprocal synapses between granule cell gemmules and mitral cell dendrites are implicated in mediating feedback and lateral inhibition of the mitral and tufted cells [121, 128].

In conclusion, based on the strong presence of filopodia and protospines and the weak presence of sessile dendritic appendages on these cells, spinogenesis in olfactory bulb granule cell seems to mainly follow the filopodial model.

## 4. Dendritic Spines and Filopodia of Intrinsic Neurons of the Cerebellar and Cerebral Cortices

Besides the projecting neurons of the cerebral and cerebellar cortices, interneurons must also be considered. Indeed, their dendrites also develop filopodia and spines but at a lower density than Purkinje or pyramidal cells.

### 4.1. Cerebellar Cortex

Besides the Purkinje cells, we have identified many other cerebellar neuronal types in Cajal's slides: granule cells (Figures 19(c) and 19(d)), basket cells (Figures 19(a)), star cells (Figures 19(b)), Golgi cells, Lugaro cells, and so forth. These cells also develop filopodia (Figures 19(a) and 19(b)) and dendritic spines, albeit at a lower density than Purkinje cells. Some dendritic spines remain in adult cells, and the spine density is dependent on the cell type. Basket and star cells have the greatest spine density followed by Golgi cells whereas Lugaro cells only occasionally have dendritic spines. It has been pointed out that these cells are smooth in the mature animal while they have filopodia during development, arguing against the role of filopodia in spinogenesis. The fact that dendrites of mature cells have few spines and that these cells develop filopodia during development, makes it possible that some of these filopodia could originate dendritic spines. It should be noted that adult granule cells do not bear dendritic spines, although their dendrites contain pedunculated appendages with head and neck. The formation of these appendages is protracted and depends on an interaction with mossy fibers. At the beginning of its development, the tip of the granule cell dendrites contains a bulb, and it may contain some dendritic filopodia. Later, some pedunculated appendages similar to dendritic spines appear whereas the dendritic terminals become more complex, forming the adult claw endings (Figures 19(c) and 19(d); supplementary videos 5 and 6).

### 4.2. Cerebral Cortex

We have also studied some interneurons in the cerebral cortex, such as the double bouquet cells (Figure 19(e); supplementary video 7), basket cells (Figure 19(f); supplementary video 8), and Martinotti cells. These cells also have spines like pyramidal cells, although the proportion of filopodia in the initial stages of development and of dendritic spines at advanced development stages is very much lower. Interestingly, protospines are also present, suggesting that the filopodial model also operates in these cells, although some sessile spines seem to emerge directly from the dendritic shaft. According to Kawaguchi et al. [129], spine density also varies in the different interneurons of the cerebral cortex (Martinotti cell, double bouquet cell, and basket cell), and many of these dendritic spines establish functional synaptic connections (70% in the Martinotti cell). In hippocampal cultures, parvalbumin-positive interneurons transfected with the GluR2 subunit of the AMPA receptor express many dendritic spines [87], suggesting that these interneurons have the potential to form dense spines on their dendrites like pyramidal cells, and that their spinogenesis is also highly regulated at the genetic level.

## 5. Towards a Unifying Model of Spinogenesis

The three models of spinogenesis proposed [14] are based on partial visions of a unique but more general model common to all regions of the nervous system. The arguments justifying a unified model of spinogenesis are summarized as follows.

**Figure 12:**
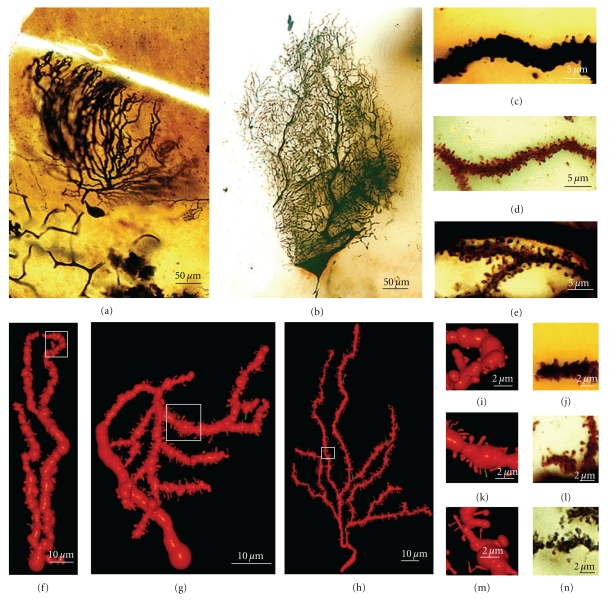
(a) Depicts a Purkinje cell, “adult bird” according to the label of the Cajal histological preparation, Golgi method. (b) Purkinje cell of “adult man” according to the label of the Cajal histological preparation, Golgi method. (c) Golgi-impregnated dendritic spines of the Purkinje cell of “adult bird.” (d) Golgi-impregnated dendritic spines of the Purkinje cell of “adult cat.” (e) Golgi-impregnated dendritic spines of the Purkinje cell of “adult man.” (f) Three-dimensional reconstruction of a portion of the dendritic tree of the “adult bird” (a). (g) Three-dimensional reconstruction of a portion of the dendritic tree of the “adult cat.” (h) Three-dimensional reconstruction of a portion of the dendritic tree of the “adult human.” (i) Boxed region in (f) showing sessile dendritic spines. (j) Golgi-impregnated sessile spines of “adult bird.” (k) Boxed region in (g) showing pedunculated spines. (l) Golgi-impregnated pedunculated spines of “adult cat.” (m) Boxed region in (h) showing pedunculated spines. (n) Golgi-impregnated pedunculated spines of “adult human.” Filopodia are color coded in green.

The model of *Sotelo* proposes that cerebellar dendritic spines emerge from dendritic trees through an autonomous cell program, independent of axon terminals (Figure 3(a)). Such a conclusion is based on the analysis of cerebellar mutants [25]. However, in normal animals, Purkinje cell branchlets grow “*long spine-like processes*” [15] that only form synaptic contacts and a terminal head after contacting parallel fibers (Figure 4(a)). In the present scientific nomenclature, these “*long spine-like processes*” can be considered as small filopodia. Thus, the synapse between the parallel fiber and dendritic spine provides a simple example of spinogenesis via filopodia and protospine (Figure 4(a)). Cajal [23] described similar small filopodia in a Purkinje cell from a 15 day-old cat (Figure 10(b)), and we found filopodia and protospines in the cerebellum in Cajal's preparations (this chapter, Figures 10, 11, and 12). Filopodia and protospines were observed by Berry and Bradley [24] in the rat cerebellum (Figure 10(d)), and Larramendi [15] found “*long synaptic adhesions*” between parallel fibers and small dendritic appendages that become mature dendritic spines through a process of “*synaptic adhesion waning*” (Figure 4(b)).

The *Miller/Peters model* is based on the maturation of the rat visual cortex (Figure 3(b)). It proposes that the formation of dendritic spines begins with a symmetric synapse between a dendrite and an axon, which gives rise to a dendritic elevation that gradually elongates and converts into a spine, at the same time the synapse becomes asymmetric. In the rat visual cortex, dendrites form synapses with symmetric densities as early as day 3 and asymmetric ones by day 9 [26]. Cotman et al. [130] described similar phases of dendritic spine formation in the dentate gyrus of the rat, as did West and Del Cerro [131] in the molecular layer of the foetal rat cerebellum, and J. W. Hinds and P. L. Hinds [132] in the mouse olfactory bulb. The latter authors described isolated postsynaptic densities that can be converted to synapses when a presynaptic specialization develops opposite to them at the beginning of synaptogenesis (E15). Indeed, they calculated that symmetric synapses take 9-10 hours to transform into asymmetric ones. However, in the visual cortex of 19-day-old mice, asymmetric synapses between a dendritic spine and axon form as the spine matures [52]. Wiens et al. [53] found that dendritic spines grow directly from the dendritic shaft, previously contacted by an axon that induced the clustering of Rac1, and subsequent morphological changes led to spinogenesis. Later on, Knott et al. [60] and Nägerl et al. [54] also found that synapses appear after the emergence of the dendritic spine in newly formed spines in the barrel cortex of adult mice *in vivo* and in CA1 pyramidal neurons in organotypic hippocampal slices, respectively

The intermediate phase in the development of a pedunculated spine is similar to that of a stubby spine (Figures 5 and 2(a)). Miller and Peters [26] suggested “*if the stubby spines are developing ones, spine formation and remodelling may be occurring even in the adult animal.*” Parnass et al. [57] found considerable morphological conversion between each category of dendritic appendages (filopodia, stubby spine, and pedunculated spine) using two-photon time-lapse imaging of developing hippocampal pyramidal neurons transfected with E-GFP in cultured slices, although the period studied was only between 2–4 h.

**Figure 13:**
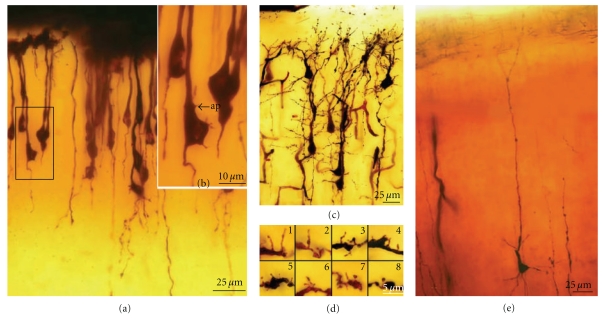
(a) Extended focus images of pyramidal cells, bipolar phase, neocortex, dog fetus, Cajal histological preparation, Golgi method. (b) Boxed region in (a) showing some appendages (ap) on the soma and the initial portion of the apical trunk. (c) Pyramidal cells, cerebral cortex, layers I–III, newborn rabbit, Cajal histological preparation, Golgi method. (d) Filopodia, protospines and dendritic spines, pyramidal cells showed in (c) 1: filopodia giving rise to protospines; 2 and 3: protospines; 4: filopodia; 5 and 6: thin dendritic spines; 7 and 8: mushroom dendritic spines. (e) Pyramidal cell, phase of appearance of basilar dendrites and collateral oblique dendrites of the apical trunk, neocortex, layer III, human fetus, Cajal histological preparation, Golgi method.

**Figure 14:**
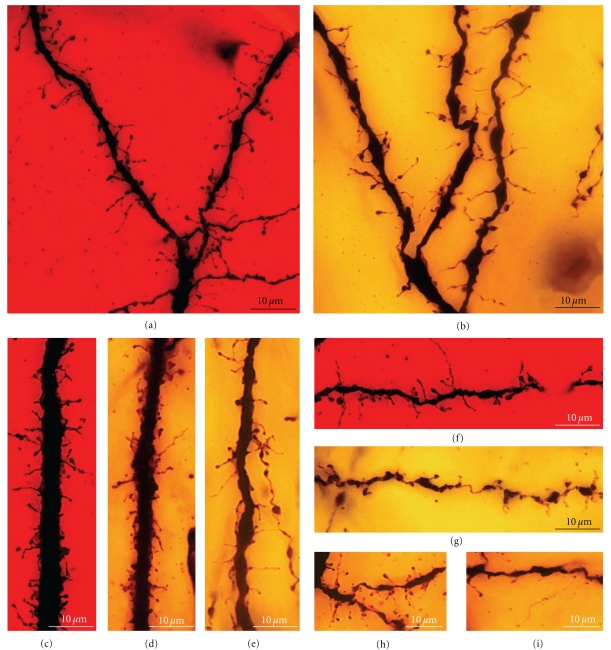
Extended focus images of filopodia, protospines and dendritic spines of the pyramidal cells from fetal and newborn animals, Cajal histological preparations, Golgi-method. (a) Apical tuft, pyramidal cell, visual cortex, fetus “almost newborn” according to the label of the Cajal histological preparation, cat. (b) Apical tuft, pyramidal cell, cerebral cortex, newborn rabbit. (c) Apical trunk, layer V pyramidal cell, visual cortex, fetus “almost newborn,” cat, bipolar phase. (d) Apical trunk, layer V pyramidal cell, newborn cat. (e) Apical trunk, layer V pyramidal cell, cerebral cortex, newborn rabbit. (f) Basilar dendrite, layer V pyramidal cell, cerebral cortex, fetus “almost newborn,” cat. (g) Basilar dendrite, layer V pyramidal cell, visual cortex, newborn rabbit. (h) Oblique dendrite, apical trunk, layer V pyramidal cell, cerebral cortex, newborn cat. (i) Oblique dendrite, apical trunk, layer V pyramidal cell, cerebral cortex, newborn human.

**Figure 15:**
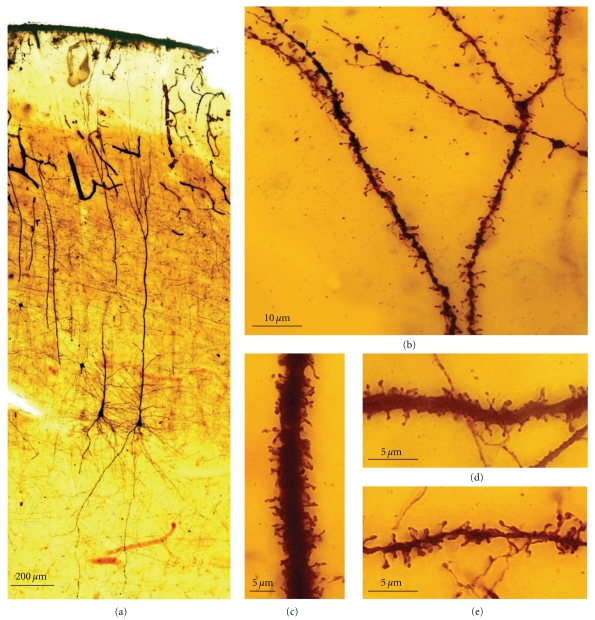
The figure represents protospines, dendritic spines, and some filopodia. (a) Layer V pyramidal cells, motor cerebral cortex, 15-day-old child, Cajal histological preparation, Golgi-method. (b) Apical tuft. (c) Apical trunk. (d) Oblique apical dendrite. (e) Basilar dendrite.

**Figure 16:**
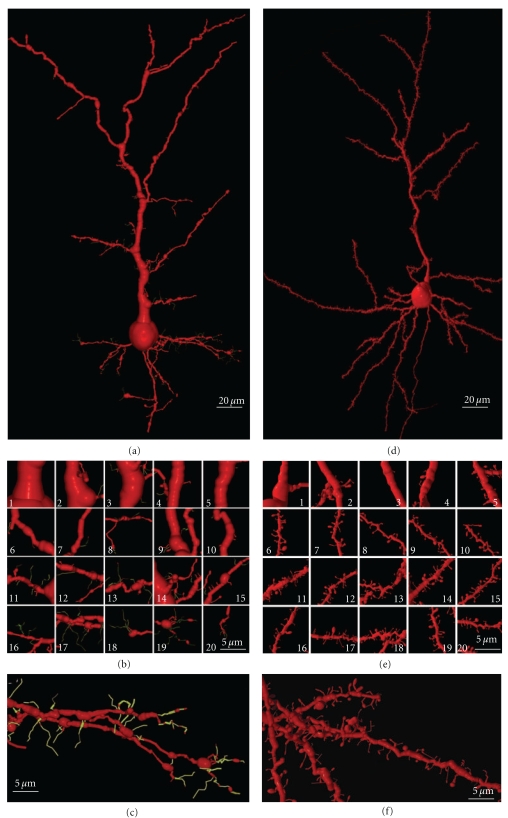
(a) Illustrates a three-dimensional reconstruction, layer III pyramidal cell, “newborn rabbit” according to the label of the Cajal histological preparation, Golgi-method. (b) Magnified parts of (a); 1–5: successive parts of the apical trunk from the soma; 6–10: parts of the oblique dendrites of the apical trunk; 11–20: parts of the basilar dendrites. (c) Magnified basilar dendrite of (a) showing collateral filopodia, protospines, and growth cone filopodia. Filopodia and protospines are color coded in green. (d) Three-dimensional reconstruction, layer III pyramidal cell, “young mouse” according to the label of the Cajal histological preparation, Golgi-method. (e) Magnified parts of (d); 1–5: apical dendrite; 6–10: oblique dendrites; 11–20: basilar dendrites. (f) Magnified basilar dendrite showing different types of dendritic spines, protospines, and filopodia.

**Figure 17:**
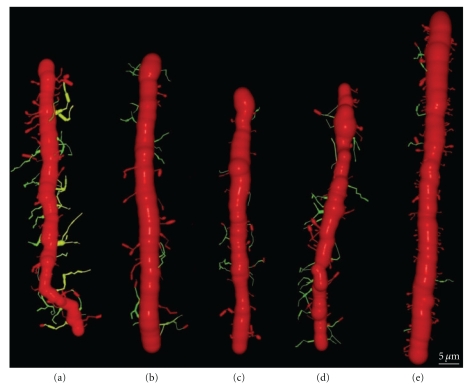
The figure depicts three-dimensional reconstructions showing the development of the apical trunk, layer V pyramidal cells, visual cortex, cat, Cajal histological preparations, Golgi-method. (a) Newborn cat. (b) 9-day-old cat. (c) 20-day-old cat. (d, e) one-month-old cat. Filopodia and protospines are color coded in green, and their number decreases with the animal age.

In the rat visual cortex, Miller and Peters [26] found filopodia between 3 to 12 days, extending from all dendrites, but rarely at days 15 and 21. Although filopodia can form synaptic contacts, they were considered transient structures not related to dendritic spine formation [26]. We found filopodia, protospines, and dendritic spines (thin and mushroom) in pyramidal cells of the newborn rabbit cerebral cortex (Figure 13(d)1–8) during developmental phase (IV). There are many dendritic filopodia, protospines and some dendritic spines (Figure 14) in the following developmental phase (V), but the ratio then reverses, many more dendritic spines appearing than dendritic filopodia and protospines (Figures 15–17). In addition, Fiala et al. [20] found dendritic filopodia forming asymmetric synaptic contacts with axons, especially during the first postnatal week in the rat hippocampal area CA1.

The *filopodial model* is based on the data gathered from developing hippocampal tissue slices (Figure 3(c)). Lateral filopodia were replaced by spine-like structures (protospines) with a slow turnover [27]. The ultimate fate of filopodia and protospines is still unclear; although it was proposed that filopodia recruit shaft synapses that later give rise to spines through a process of outgrowth [20]. Larramendi [15] interpreted his data as if filopodia could give rise to dendritic spines (Figure 4), and Parnass et al. [57] confirmed that most filopodia produce sessile and pedunculated spines, although filopodia could also extend out of existing spines. De Roo et al. [103] found that filopodia only occasionally lead to the formation of stable dendritic spines by enlargement of the spine head and the expression of PSD-95 within a critical period of 24 h.

The *unifying model of spinogenesis* that we propose takes into account the development of the neuron and the asynchrony and plasticity in the formation of dendritic spines. The following modes or strategies can be distinguished.

**Mode 1**

At the very beginning of synaptogenesis (E15, mouse [132]), isolated postsynaptic densities can convert to synapses when a presynaptic specialization develops opposite them. The initial symmetric contact is transformed into an asymmetric one by 9-10 hours [132]. The outgrowth of these contacts gives rise to the first dendritic spines. During the maturation of the dendritic spine, the length of the synaptic contact diminishes, a process called “*synaptic adhesion waning*” [15]. In this embryonic period, the first axons arriving could easily contact with the dendrites because there are abundant spaces in the neuropil.

**Mode 2**

In newborn animals and during the first postnatal week, lateral dendritic filopodia can develop varicosities and heads giving rise to multispines [99] or protospines [27] after contacting with axons. In this developmental period, the intercellular spaces in the neuropil diminish, and the emission of filopodia could be a neuronal strategy to capture and recruit axonal endings to new dendritic spines (see [15, 20, 68, 69]) and at the same time increasing the dendritic capture volume [18]. Filopodia contacting several axons can choose the most active ones and, perhaps, discriminate between distinct types of axonal endings.

**Mode 3**

In young [52, 19-day-old mouse] and adult animals [60] and also in organotypic hippocampal slices [19, 27, 54, 56, 57], young dendritic spines contacting with axons do mature at the same time as that of the synaptic contacts.

These three modes or strategies to form dendritic spines have a specific temporal window, but with a considerable overlap and a characteristic intensity peak. In addition, the development of dendritic structures (spines, filopodia and protospines) is asynchronous. Thus, we can distinguish distinct phases of maturation of these structures, which also makes it a little more difficult to interpret the experimental results. Even in the adult mouse neocortex, 3.6% of the spines clearly lacked synapses, and they resembled small dendritic filopodia [108].

## 6. Future Directions

Dendritic spines can develop without the assistance of filopodia. In this regard, what is the role of dendritic filopodia? Some authors have speculated about the role of filopodia as a bridging structure to facilitate axodendritic synaptic contacts, increasing the dendritic capture volume. Current data corroborated the implication of filopodia in synaptogenesis and their eventual transformation into dendritic spines. However, the cellular and molecular mechanisms of this transformation are still unclear. Are the filopodial synapses incorporated into the dendrite shaft prior to the spine growing? Do contacting axons follow the incorporation of filopodial synapses to the dendritic shaft?

The meaning of the intermediate phase between filopodia and dendritic spines (multispine or protospine) is still uncertain. The question arises as to whether this phase is necessary to discriminate active axonal endings when there is a period of large-scale arrival of specific axons. Alternatively, could multispines also be implicated in sensing and/or integrating different active axons prior to moving them to the dendritic shaft? 

New improvements in microscopy, such as stimulated emission depletion (STED) microscopy, with greater resolution than confocal or two-photon microscopy, will allow us to visualise activity-driven structural changes of spines with unprecedented clarity. This technique should reveal fine details such as the shape of the spine head or the width of the spine neck [133], which together with the development of visualization methods showing complete axons will help advance studies of the interaction of axons with filopodia, protospines, and spines. Further studies are needed to determine the mechanisms implicated in the initial formation of dendritic spines at the very beginning of synaptogenesis during embryogenesis (mode 1 of the unifying model), as well as the possible influence and/or the shared mechanisms in the development of lateral filopodia (mode 2) and dendritic spines in young and adult animals (mode 3).

High-frequency focal synaptic stimulation by activation of NMDA receptors, overexpression of GluR2 and TM-agrin, proteolytic cleavage of agrin by neurotrypsin, or activation of SK3 channel, all induce filopodia growth. However, more studies are needed to better understand the mechanisms of filopodia formation and retraction. 

Clustering of Rac1 has been implicated in the formation of spines directly from the dendritic shaft previously contacted by an axon, recruiting AMPA receptors to synapses and providing a link between presynaptic and postsynaptic developmental changes. A basic mechanism to form stable dendritic spines is the expression of PSD-95 within a critical period of 24 h. This protein may help control synapse formation by regulating nNOS expression at the synapse, thereby promoting NO release and/or activation of cGMP signalling in presynaptic axons. However, the details of activity-dependent growth and evolution of dendritic spines related to learning and memory are far from clear.

## Supplementary Material

**Figure 18:**
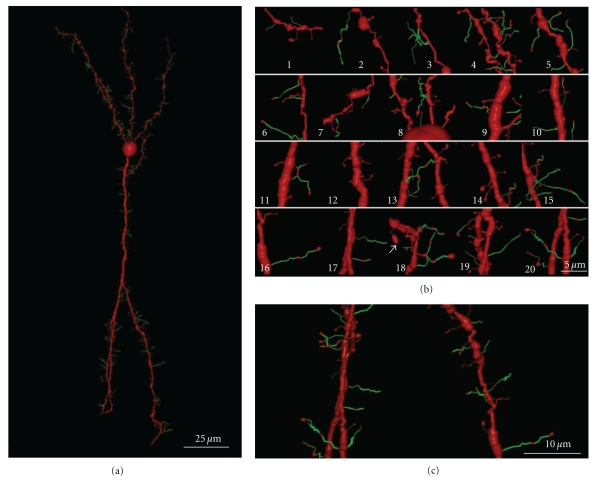
(a) Depicts three-dimensional reconstructions, granule cell, olfactory bulb, one-month-old dog, Cajal histological preparation, Golgi-method. (b) Filopodia, protospines and spines; 1–10: inner dendrites (above the soma); 11–15: peripheral dendrites (below the soma); 16–20: peripheral apical tuft. (c) Filopodia, protospines and spines of the peripheral apical tuft. Filopodia and protospines are color coded in green. The arrow in (b) 18 points to a very big head with long neck possibly a *gemmule*, a characteristic appendage of this type of cell.

**Figure 19:**
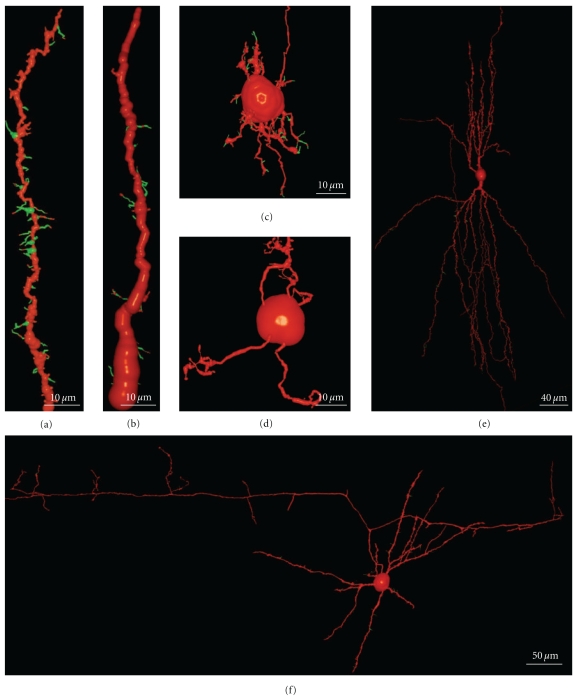
The figure shows filopodia, protospines, and dendritic spines of intrinsic neurons of the cerebellar and cerebral cortices, three-dimensional reconstructions, Cajal histological preparations, Golgi-method. (a) Basket cell dendrite, cerebellum, 17-day-old dog. (b) Golgi cell dendrite, cerebellum, 17-day-old dog. (c) Young immature granule cell, cerebellum, newborn cat. (d) Mature granule cell, cerebellum, adult cat. (e) Double bouquet cell, motor cortex, layer III, one-month-old child. (f) Basket cell, motor cerebral cortex, layer IV, one-month-old dog. Filopodia and protospines are color coded in green.

Video 1 is a moving 3D–model showing the possible role of filopodia in the processes of synaptogenesis and spinogenesis.
Seven videos illustrate and complement the descriptions and figures of the text by means of rotation, translation and scaling of 3D–reconstructions made from optical serial sections of the histological preparations of Santiago Ramón y Cajal:
Video 2 shows the figure 16 (a): a layer III pyramidal cell of a newborn rabbit; filopodia and protospines are color coded in green.
Video 3 shows figure 16(d): layer III pyramidal cell of a young mouse. The last frames of video 2 and video 3 show a comparison of the 3D–image obtained using the Golgi method and the stack of images of living tissue using two–photon confocal microscopy.
Video 4 depicts figure 18(a): a granule cell of the olfactory bulb, one–month–old dog. Filopodia and protospines are color coded in green.
Video 5 shows figure 19(c): a young immature granule cell of the cerebellum, newborn cat. Filopodia and protospines are color coded in green.
Video 5 shows figure 19(c): a young immature granule cell of the cerebellum, newborn cat. Filopodia and protospines are color coded in green.
Video 7 shows figure 19(e): a double bouquet cell of the motor cortex, layer III, one–month–old child. Filopodia and protospines are color coded in green.
Video 8 shows figure 19(f): a basket cell of the motor cerebral cortex, layer IV, one–month–old dog. Filopodia and protospines are color coded in green.

## Abbreviations

AMPA: *α*-amino-5-hydroxy-3-methyl-4-isoxazole propionic acid
APV: DL-2-amino-5-phosphonovaleric acid
CA1: cornu ammonis 1
cGMP: cyclic guanosine monophosphate
DIV: days in vitro
GABA: *γ*-aminobutyric acid
GFP: green fluorescent protein
GluR2: glutamate receptor R2
MAPK: mitogen-activated protein kinase
NMDA: N-methyl D-aspartate
nNOS: neuronal nitric oxide synthase
NO: nitric oxide
PSD: post-synaptic density
Rac1: Ras-related C3 botulinum toxin substrate 1
Ras: Rat sarcoma
SK3 channel: small-conductance calcium-activated potassium channel
TBS: electrical theta-burst stimulation
TM-agrin: transmembrane form of agrin.

## Endnotes

1. Some of Cajal's works translated into English are: (i) Javier DeFelipe and Edward G. Jones, (1991). *Cajal's Degeneration and Regeneration of the Nervous System*. Translated by Raoul M. May. Edited by DeFelipe and Jones. Oxford University Press. (ii) Javier DeFelipe and Edward G. Jones, (1988). Cajal on the Cerebral Cortex. Translated by DeFelipe and Jones. Edited by Corsi, Jones and Shepherd. Oxford University Press.
2. “*… the surface … appears bristly with tips or short spines …”* [1].
3. “… we show in figure 50 several drawings taken from the shafts of young or adult cerebral pyramids. *A* represents the shaft of a pyramid from the visual region of a nearly mature rabbit. Note how short the spines are and how they begin very thin and end by a terminal bulb. Very few of them are bifurcated. In *C* the shaft of a pyramid of a one-month-old cat is shown. Here the arrangement described in *A* is confirmed; the spines appear a little bit longer and are frequently curved. In *B* (two-months-old child) I have drawn another shaft of a pyramid of the visual region. Here not only the greater length of the appendages but also the frequency with which they divide and change the direction of their secondary branchlets call attention. As means of comparison we have drawn the dendrite of a motor cell of the spinal cord (*D*, one month old cat). Observe that the surface is bristling with irregular projections very seldom ending as bulbs. It is almost certain that this arrangement is transitory. Finally, we have drawn several cruciform or oblique nerve collaterals of the cerebrum. It is impossible to see any fusion of the latter with the spines.”
4. “... it is not rare to see in the terminal portion or base, a longer membranous process, a sort of protoplasmic outpost between intercellular or interepithelial interstices.”
5. According to the labelling of Cajal's histological slide.
6. “Almost all their branches (Figure  2(b), Figure  163 of this work) possess mild spines perpendicularly inserted into their contour. These spines appear to be dyed a clear coffee colour and they are bigger than those of the terminal twigs of the adult corpuscles.”
7. “...the peripheral expansion of the granular cells has an unchanged orientation and connection, since it will constantly runs to the plexiform zone, where it terminates in favor of a tuft of very spiny branches, in contact with the secondary dendrites born in the mitral cells.”
